# Unexpected cure of a toxic nodule in a multinodular goiter induced by immune checkpoint inhibitors: a case report

**DOI:** 10.1530/ETJ-22-0024

**Published:** 2022-05-26

**Authors:** Hippolyte Dupuis, Emilie Merlen, Arnaud Jannin, Philippe Jamme, Alexandre Fagart, Marie-Christine Vantyghem, Miriam Ladsous

**Affiliations:** 1 Department of Endocrinology, Diabetology and Metabolism, Huriez Hospital, Lille University Hospital, Lille, France; 2 University of Lille, Lille, France; 3 Department of Dermatology, Lille University Hospital, Lille, France; 4 Department of Nuclear Medicine, Valenciennes Hospital Center, Valenciennes, France

**Keywords:** thyroid atrophy, immune checkpoint inhibitors, toxic nodule, multinodular goiter, destructive thyroiditis

## Abstract

**Introduction:**

Immune checkpoint inhibitors (ICI) are used to treat cancers including metastatic melanomas and can induce endocrine side effects. The thyroid is frequently affected with classically transient thyrotoxicosis followed by hypothyroidism. The evolution of thyroid nodules and goiters under ICI therapy is poorly described.

**Case presentation:**

A 72-year-old male presenting with hyperthyroidism due to a toxic nodule in a multinodular goiter (MNG) started ICI therapy combining ipilimumab and nivolumab to treat metastatic melanoma. After an initial worsening of thyrotoxicosis, treated with carbimazole, he developed profound hypothyroidism, persisting after carbimazole discontinuation, needing a long-term levothyroxine supplementation. Ultrasound control performed 6 months after ICIs treatment initiation revealed diffuse thyroid atrophy with involution of all nodules. ^123^I-scintigraphy confirmed a destructive mechanism.

**Discussion:**

The evolution of MNG and toxic nodules is poorly described in patients treated with ICI since systematic US evaluations are lacking. We describe for the first time a toxic nodule cured by ICI therapy inducing destructive thyroiditis.

**Conclusion:**

Pre-existing nodules and MNG, even if toxic, are not a contraindication for ICI treatment provided the patients are carefully monitored.

## Established facts

- Immune checkpoint inhibitors (ICI) can induce endocrine side effects, the most common of which is thyroid dysfunction.
- The main pathological mechanism of ICI-induced thyroid dysfunction is destructive thyroiditis, which may lead to thyroid atrophy.

## Novel insights

- ICI-induced destructive thyroiditis can cause a significant volume reduction of goiter and thyroid nodules and inactivation of toxic nodules.
- Pre-existent nodule or multinodular goiter, even if toxic, should not necessarily be a contraindication for ICI treatment.

## Introduction

Immune checkpoints are crucial in the regulation of the immune system and self-tolerance. Immune checkpoint inhibitors (ICIs) are monoclonal antibodies that promote the activation of the immune system against cancer. Molecules using monoclonal antibodies against the cytotoxic T lymphocyte-associated protein 4 (CTLA-4), such as ipilimumab, and against the programmed cell death 1 receptor (PD-1), such as nivolumab or pembrolizumab, have significantly modified the prognosis of metastatic melanomas, as well as renal and lung cancers (1). ICI can be responsible for immune responses against physiological tissues that can induce damage in various organs including the endocrine glands. In addition, the combined use of monoclonal antibodies against CTLA-4 and PD-1 significantly increases the risk of adverse events (36% of rate) (2).

Thyroid damages are the most frequent endocrine side effects. The 2018 French expert opinion reported a 3–16% incidence of thyrotoxicosis and 3–22% occurrence rate of hypothyroidism; the overall rate of thyroid dysfunctions could reach 50% including the rough forms (3). The classic presentation is a silent inflammatory destructive thyroiditis mediated by T-cell cytotoxicity. It includes an initial transient and inconsistent thyrotoxic phase occurring between the third and the sixth week of treatment, followed by overt or subclinical hypothyroidism occurring between the sixth and tenth week of treatment. The current guidelines recommend to symptomatically treat and monitor transient thyrotoxicosis and to supplement overt hypothyroidism with levothyroxine (3, 4).

## Case report

**Figure 1:**
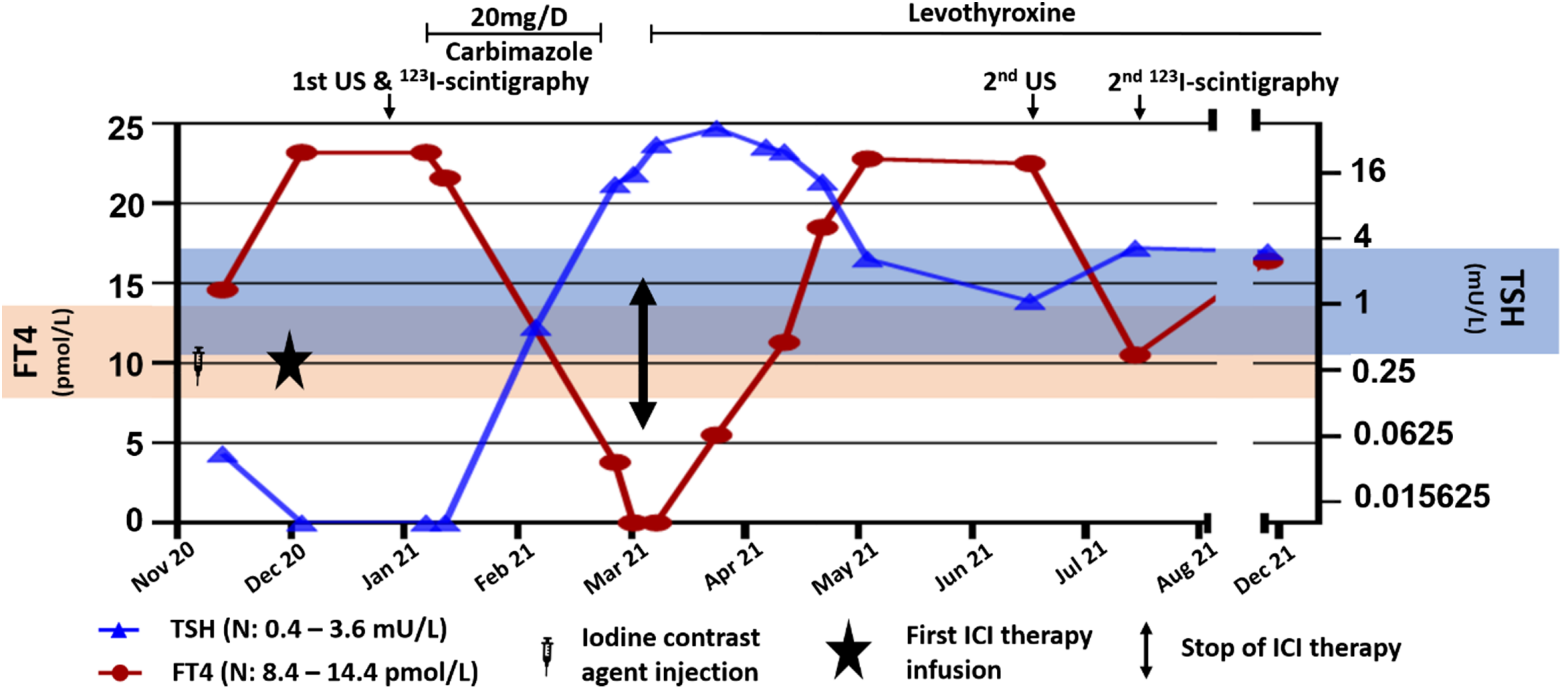
Evolution of thyroid function, treatment periods and dates of morphological and functional examinations.

**Figure 2:**
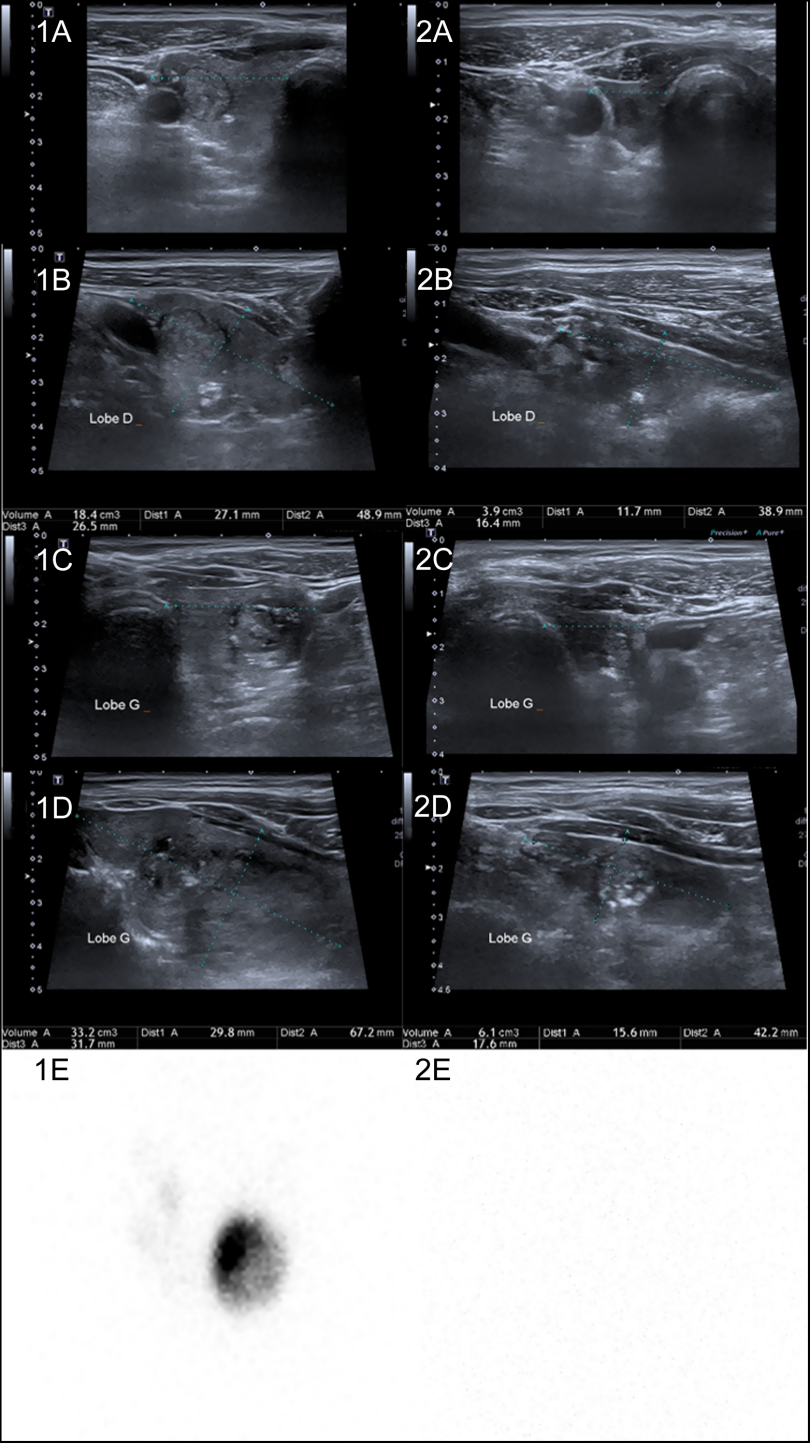
Thyroid ultrasound and ^123^I-scintigraphy imaging before (left column) and after (right column) ICI therapy. (1a and 1b) Transverse and longitudinal US images of the right thyroid lobe before ICI therapy, assessing a 18.4 mL volume and three nodules. (1c and 1d) Transverse and longitudinal US images of the left thyroid lobe before ICI therapy, assessing a 33.2 mL volume and two nodules. (2a and 2b) Transverse and longitudinal US images of the right thyroid lobe 7 months after ICI therapy initiation, assessing a 3.9 mL volume and no visible nodule (except for an isolated macrocalcification). (2c and 2d) Transverse and longitudinal US images of the left thyroid lobe 7 months after ICI therapy initiation, assessing a 6.1 mL volume and only one visible nodule. (1e) ^123^I-scintigraphy 13 days after the first ICI infusion showing a left lobe heterogeneous hot nodule and suppressed uptake in the rest of the thyroid. (2e) ^123^I-scintigraphy 7 months after ICI therapy initiation showing absent ^123^I uptake.

We report a 72-year-old male who was incidentally found to have mild hyperthyroidism with low TSH (0.043 mU/L (0.4–3.6)) and slightly increased free thyroxine (FT4) (14.6 pmol/L (8.4–14.4)) (Fig. 1). Free triiodothyronine (FT3) was not measured. The patient had received an iodine-contrast injection for a CT scan 10 days before. He did not take any other treatment interfering with the thyroid function, had no fever, no local anterocervical pain and anti-thyrotropin-receptor antibodies were negative. Ultrasound (US) examination revealed a 51.6 mL multinodular goiter (MNG) with three main nodules in the right lobe (one 15 mm EU-TIRADS III nodule and two EU-TIRADS IV micronodules) and two nodules in the left lobe (25 mm EU-TIRADS V in the middle part and 33 mm EU-TIRADS III in the lower part) (Fig. 2, 1a to 1d). Fine-needle aspirations (FNA) of these two-left lobe macronodules showed Bethesda III and Bethesda I lesions, respectively.

Meanwhile, the patient was diagnosed as having pulmonary and lymph nodes metastasis of a superficial melanoma of the calf, Breslow 1.8 mm, *BRAF* non-mutated, operated on 8 years earlier. ICIs associating ipilimumab and nivolumab were started on 2 weeks after the diagnosis of hyperthyroidism. Two days after the first ICI infusion and 6 weeks after the iodine injection, thyrotoxicosis worsened: (FT4, 23.2 pmol/L and FT3, 6.5 pmol/L (3.8–6)). ^123^I-scintigraphy, performed 13 days after ICI initiation, showed a heterogeneous hot nodule of the lower part of the left thyroid lobe, contrasting with suppressed uptake in the rest of the gland, including the other nodules (Fig. 2, 1e). In line with timing consideration, the scintigraphy suggested that the worsening of hyperthyroidism was rather related to an increased nodular hormonal production favored by the recent iodine injection than to ICI-induced destructive thyroiditis. Carbimazole 20 mg/day was initiated and nivolumab alone was continued after the third double-agent infusion because of a cutaneous adverse event (skin rash with hypereosinophilia).

Six weeks after ICI initiation, TSH increased (15 mU/L) and FT4 was undetectable. Carbimazole was stopped but TSH continued to raise (29 mUI/L), FT4 remained undetectable and FT3 was low (3 pmol/L). Levothyroxine replacement therapy was initiated, at a dose of 1.3 µg/kg/day, and gradually increased to 1.8 µg/kg/day since TSH was persistently elevated (41 mU/L). Anti-TPO antibodies were positive (265 U/mL (*n* < 7)) and anti-thyroglobulin antibodies were negative. Nivolumab was withdrawn 3 months after initiation because of persistent skin toxicity. TSH completely normalized 4 months after the initiation of the levothyroxine treatment. At that time (5 months after ICI initiation), a new US thyroid evaluation showed marked thyroid atrophy: the right lobe was 3.9 mL and the left lobe 6.1 was mL (vs 18.4 and 33.2 mL, respectively, initially). Parenchyma was highly hypoechoic and avascular. In the right lobe, the nodules had disappeared: only a 6 mm isolated macrocalcification was noted. In the left lobe, only one of the two nodules was identified, in the middle part of the lobe, with a maximum diameter reduced to 14 mm (vs 25 mm at the first evaluation), classified as EU-TIRADS V (Fig. 2, 2ato 2d). The toxic nodule was no longer visible in the lower part of the lobe. The plasma thyroglobulin was undetectable, without any anti-thyroglobulin antibody, and ^123^I-scintigraphy showed absent thyroid uptake, confirming the probable destructive mechanism of thyroid dysfunction (Fig. 2, 2e). The patient had not performed any CT scan with iodine contrast agent in the last 8 months. One year after ICI initiation, hypothyroidism persisted and US was not further modified.

**Figure 3:**
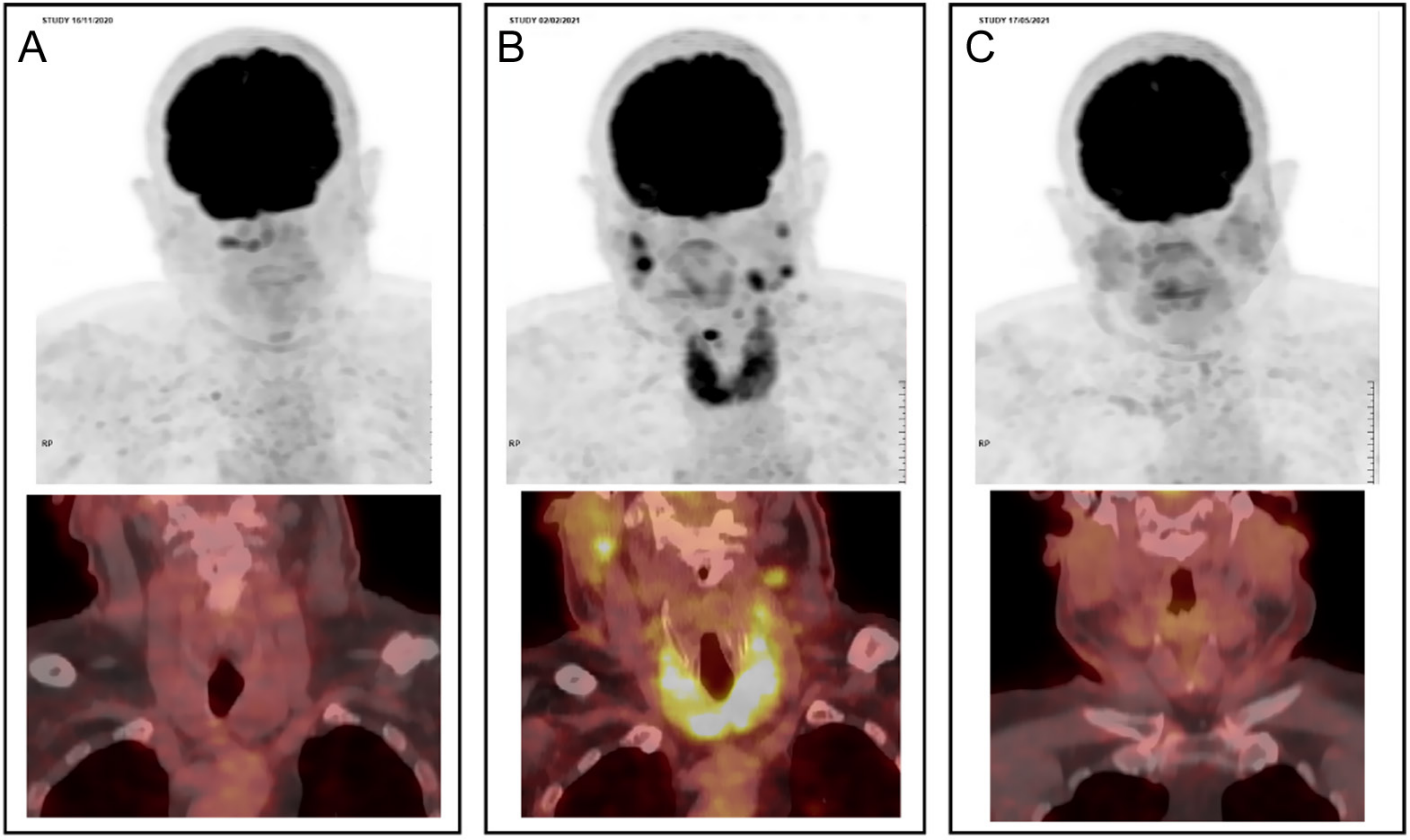
(A) ^18^F-FDG PET/CT before ICI initiation (November 2020) showed absent thyroid hypermetabolism. (B) 2 months after ICI initiation (February 2021), an intense and diffuse ^18^F-FDG uptake was observed in the thyroid gland (SUV_max_ 9.5). (C) ^18^F-FDG PET/CT performed in May 2021, after ICI withdrawal, showed regression of thyroid ^18^F-FDG uptake and thyroid atrophy.

Of note, ^18^F-FDG PET/CT did not show any significant thyroid uptake before initiation of ICIs. By contrast, an increased and diffuse ^18^F-FDG uptake (SUV_max_ 9.5) was observed in the thyroid gland under ICI treatment and then regressed after ICI withdrawal, coinciding with thyroid atrophy and hypothyroidism (Fig. 3). Elevated uptake of several cervical lymph nodes was associated with thyroid uptake, suggesting an activation of the immune system. A complete response of the pulmonary solitary metastasis was observed 9 months after initiation of ICIs, after additional stereotaxic radiotherapy with CyberKnife.

## Discussion

We describe for the first time the outcome of a toxic nodule in a MNG under ICI treatment. This treatment induced destructive thyroiditis with parenchymal atrophy and regression of nodules at US evaluation and led to the remission of thyrotoxicosis and onset of hypothyroidism. US evaluation and ^123^I-scintigraphy showed that the entire MNG, including the toxic nodule, was affected by the destructive thyroiditis process. The only persistent nodule was an EU-TIRADS V, Bethesda III nodule of left lobe, maximum diameter of which decreased from 25 to 14 mm. We cannot exclude that this nodule corresponds to a thyroid carcinoma, which may be less sensitive to ICI than other benign nodules. The patient refused, so far, a new FNA and/or thyroidectomy.

Although the biological monitoring of thyroid function before and during the use of ICI is now recommended by guidelines, US examination of the thyroid is rarely performed, and few studies reported both baseline and follow-up US patterns. In a cohort of 52 ICI-treated patients, 6 patients had a MNG before therapy initiation, 3 of them were initially thyrotoxic, whereas 2, who were initially euthyroid, developed ICI-induced thyrotoxicosis (5). However, the evolution of thyroid function tests of the initially thyrotoxic patients, and the changes in US findings, was not reported. Only few cases of ICI-induced thyroid atrophy have been published: one was concomitant to pancreatic atrophy (6), whereas an initial hypoechogenic thyroid enlargement was reported in 3 other patients who developed destructive thyroiditis, followed by a decrease in thyroid size in one of them in a prospective cohort of 66 nivolumab-treated patients (7). Thyroid atrophy following destructive thyroiditis could be underestimated due to the lack of systematic US long-term follow-up.

^123^I- or ^99m^Tc-scintiscans are used to determine the mechanism of the transient thyrotoxicosis (7, 8). Here, the first ^123^I-scintigraphy, performed 13 days after the first ICI infusion, was too early to reflect the ICI effect on thyroid function. Indeed, ICI-induced thyroid damages usually occur after two to four ICI infusions, which means 3–12 weeks after the first infusion (9). This first scintigraphy was performed 6 weeks (45 days) after the iodine injection. It is known that urinary iodine excretion normalizes in 50% of cases 43 days after a contrast CT scan and within 75 days in 90% (10). In our patient, a persistent iodine overload could have explained the extinction of extranodular parenchyma but not the elevated uptake of the left lobe nodule. We can therefore assume that in our patient, the iodine overload had probably already disappeared at the time of the scintigraphy and that the nodule was already toxic before the iodine injection, which nevertheless contributed to the worsening of its hyperfunction. The second ^123^I-scintigraphy, performed 7 months after the ICI initiation, during the hypothyroid phase, confirmed its destructive mechanism.

It remains unclear if pre-existing thyroid antibodies play a role in the development of thyroiditis or eventual hypothyroidism under ICI treatment, mainly because of the lack of available baseline values (7, 8, 11). Here, TPO-Abs were found positive after the initiation of ICI, but baseline dosage was not available. In one study, authors observed that HLA-DPA1*01:03 and DPB1*02:01 were more frequent in patients with ICI-induced hypothyroidism requiring hormone supplementation for more than 3 months compared to a control group (12). However, there is currently no HLA typing to predict the risk of thyroid atrophy under ICI.

Other predictive factors of ICI-induced thyroid atrophy could be studied such as the association with other adverse events or the RECIST response of secondary lesions. ^18^F-FDG uptake of thyroid gland during ICI treatment could also be a reliable tool to predict thyroiditis with subsequent hypothyroidism (13).

The biological thyroid function was regularly monitored. Although TSH was stable under levothyroxine 150 µg/day, the FT4 levels fluctuated widely. Our main hypothesis is that there may be immunoassay interferences induced by nivolumab, which have been already described (14). This could explain that TSH did not decrease in spite of FT4 peaks observed in May, June and December 2021. However, nivolumab was already stopped at that time, suggesting more a possible persistent immunological effect, such as nivolumab-induced anti-T4 auto-antibodies, than a biochemical mechanism. The patient did not exhibit any associated corticotropic insufficiency. However, an associated thyrotropic insufficiency could not explain this conflicting pattern. Also, we cannot exclude a fluctuating patient adherence to levothyroxine therapy.

The thyroid US will continue to be monitored, particularly the EU-TIRADS V nodule in the left lobe. If the regression stops or if a nodule regrowth is observed, a new FNA should be performed, and thyroid surgery may be considered. Long-term follow-up is also necessary to detect a possible recurrence of thyrotoxicosis.

To conclude, this observation suggests that pre-existing nodules or MNG, even if toxic, is not a contraindication for ICI treatment, provided the patient is carefully monitored. Besides, in some cases, ICI treatment could even be beneficial for thyroid pathology, avoiding thyroid surgery. Current guidelines and expert opinion on the management of ICI adverse events recommend neck US and thyroid scintigraphy only in selected cases. These evaluations seem particularly useful in case of pre-existing goiter or thyroid dysfunction. Finally, ^18^F-FDG PET/CT could be a reliable tool to predict thyroid ICI toxicity.

## Declaration of interest

The authors declare that there is no conflict of interest that could be perceived as prejudicing the impartiality of this case report.

## Funding

This work did not receive any specific grant from any funding agency in the public, commercial, or not-for-profit sector.

## Statement of ethics

Written informed consent was obtained from the patient for publication of this case report and any accompanying images.

## Author contribution statement

H D and M L wrote the manuscript. E M, A J, P J, A F and M C V contributed to the enrichment of the manuscript by their suggestions and remarks. All authors approved the final version.
